# Factor Structure and Psychometric Properties of the Family Communication Scale in the Chinese Population

**DOI:** 10.3389/fpsyg.2021.736514

**Published:** 2021-11-12

**Authors:** Ningyuan Guo, Henry C. Y. Ho, Man Ping Wang, Agnes Y. Lai, Tzu Tsun Luk, Kasisomayajula Viswanath, Sophia S. Chan, Tai Hing Lam

**Affiliations:** ^1^School of Nursing, University of Hong Kong, Hong Kong, Hong Kong SAR, China; ^2^Department of Psychology and Centre for Psychosocial Health, The Education University of Hong Kong, Hong Kong, Hong Kong SAR, China; ^3^Department of Social and Behavioral Sciences, Harvard T. H. Chan School of Public Health, Boston, MA, United States; ^4^Center for Community-Based Research, Dana-Farber Cancer Institute, Boston, MA, United States; ^5^School of Public Health, University of Hong Kong, Hong Kong, Hong Kong SAR, China

**Keywords:** family communication scale, positive family communication, communication method, information and communication technologies, validation, Chinese

## Abstract

**Purpose:** To evaluate the factor structure and psychometric properties of the 10-item Family Communication Scale (FCS) in the Chinese population.

**Methods:** Study 1 was a population-based survey [*N* = 687, 61.1% female; mean age (SD) 56.6 (19.1)]. Study 2 was a community-based intervention (*N* = 1983, 76.7% female; 57.8% aged 20–59 years). We conducted exploratory factor analysis (EFA) in Study 1 and replicated the model by confirmatory factor analysis (CFA) in Study 2. Psychometric properties were evaluated, including internal consistency, test–retest reliability, convergent and discriminant validity, and known-group validity. We identified how the FCS scores differed by sociodemographic characteristics and communication methods including face to face and Information and Communication Technologies (ICTs) in Study 1.

**Results:** The EFA and CFA supported a one-factor structure. The Chinese FCS showed a good internal consistency (Cronbach’s alpha = 0.91; McDonald’s Omega = 0.91) and was stable over 1-month (intraclass correlation coefficient = 0.69, *P* < 0.001). Convergent validity was supported by positive correlations of FCS with the Subjective Happiness Scale, Family Adaption, Partnership, Growth, Affection, Resolve (APGAR) Scale, family health, harmony, and happiness, and perceived family communication sufficiency and quality (All *P* < 0.001). Discriminant validity was supported by the stronger correlation of FCS with Short Form-12 Health Survey Version 2 Mental Component than that with Physical Component (*P* < 0.001). Higher household income, frequent face-to-face communication, and frequent use of phone calls, instant messaging, and social networking sites were associated with higher FCS scores.

**Conclusion:** The one-factor structure of the Chinese FCS can be a reliable and valid measurement of positive family communication, in the context of ICT integration into family communication.

**Clinical Trial Registration:** [www.ClinicalTrials.gov], identifier [NCT02563613].

## Introduction

Family communication is the act of sharing ideas, participating in decision making, and expressing feelings among members as a family unit (Olson, 2000). Less family communication or more family conflicts were associated with higher risks of behavioral problems such as substance use disorders and gaming disorders in young people (Challier et al., 2000; Schneider et al., 2017). In contrast, positive family communication, including aspects of listening, speaking, self-disclosure, clarity, continuity tracking, and respect may improve physical and mental health (Olson, 2000), through social support and adaptive coping strategies with stressors (Schrodt et al., 2008). Positive family communication also facilitates a balanced level of family flexibility to change family rules and cohesion of emotional bonding (Olson, 2000). These benefits on families were shown in our previous interventions indicating improved family well-being through enhancing family communication (Ho et al., 2017, 2018; Shen et al., 2017a).

The Family Communication Scale (FCS) is a widely used measurement of the satisfaction toward the aspects of positive communication among family members (Olson and Barnes, 2004), which was adapted from the 20-item Patient-Adolescent Communication Scale (PAC) measuring communication in families with adolescents (Barnes and Olson, 1985). Compared with PAC, the shorter 10-item FCS has a lighter operation burden on respondents and can be used in broader family forms and families at various life cycle stages (Olson and Barnes, 2004). The FCS has been widely used globally with consistent satisfactory reliability and validity, but only a few have reported the factor structure (Olson and Barnes, 2004; Baiocco et al., 2013; Koutra et al., 2013; Gomes et al., 2017; Martínez-Pampliega et al., 2017). A validation study in Turkey showed a one-factor structure that discarded items on self-disclosure and affective communication (Türkdoğan et al., 2018), which was controversial with the original scale having all 10 items in one factor (Olson and Barnes, 2004). Such a variable FCS scoring structure can be explained by cultural differences in family communication patterns across different populations. Unlike an expression of self-emphasized in the West, implicit communication and listening-centeredness are often used in the Asian collectivist culture (Bond, 2010). Apart from cultural differences, our previous qualitative studies in Chinese showed that family communication could be affected by interaction time, income, and psychosocial capitals (Chan et al., 2011; Lam et al., 2012).

The evolving Information and Communication Technologies [ICTs; e.g., mobile phone, instant messaging (IM), social networking sites (SNS)] have transformed communication patterns (Carvalho et al., 2015). Family communication can be conducted in real-time and/or asynchronously using ICTs, which may overcome time and distance barriers. ICTs have enabled transnational family communication for low-income immigrant families to maintain virtual intimacy, emotional support, and transnational caregiving in a qualitative interview (Gonzalez and Katz, 2016). Both factual and emotional information can be exchanged among family members through multimedia on ICTs such as texts, pictures, audio clips, and videos (Carvalho et al., 2015). Higher levels of family well-being have been observed in people who frequently used ICTs for family communication such as phone calls and video calls in our previous population-based studies (Wang et al., 2015; Shen et al., 2017b). Instruments such as Mobile Device Proficiency Questionnaire, Computer Proficiency Questionnaire (Moret-Tatay et al., 2019), and ICT competence scale (Aesaert et al., 2014) were developed for measuring ICT use and showed cross-cultural differences. Direct measures of family communication using ICTs are lacking particularly in the Chinese population.

The study aimed to evaluate the Chinese version of FCS in a population-based telephone survey sample and a community-based randomized controlled trial sample of Hong Kong Chinese. The factor structure and psychometric properties of FCS have yet to be examined in the Chinese population, compared with other validated instruments such as Family Adaption, Partnership, Growth, Affection, Resolve [(APGAR) Scale; Chan et al., 1988], the single item of family happiness in Family Well-being Scale (Shen et al., 2019). We also took advantage of the representative survey sample to identify how FCS scores differed by sociodemographic characteristics and family communication methods, including ICTs and face to face.

## Materials and Methods

### Study Design

#### Study 1: The Hong Kong Family and Health Information Trends Survey

The FHInTS is a periodic territory-wide telephone survey on information use, health communication, and family well-being among Hong Kong residents aged 18 years or above. We have conducted five waves of FHInTS since 2009 and reported details of the study design elsewhere (Wang et al., 2015; Shen et al., 2017b). Study 1 is part of the fifth wave of FHInTS, conducted from February to August 2017.

#### Study 2: Happy Family Kitchen Movement Project

The HFKM was a community-based intervention program conducted from January 2015 to July 2017 in Hong Kong residents aged 12 years or above to improve family well-being using the positive psychology framework integrated with physical and psychosocial health. Details of the study design and sociodemographic characteristics of the participants were reported elsewhere (Ho et al., 2019). The study was registered with ClinicalTrials.gov (NCT02563613).

### Participants

#### Study 1: The Hong Kong Family and Health Information Trends Survey

We used a two-stage probability-based sampling procedure. First, landline telephone numbers were randomly generated using known prefixes assigned to telecommunication service providers under the Numbering Plan provided by the Government Office of the Communications Authority. Invalid numbers were removed according to the computer and manual dialing records. Telephone numbers of respondents from previous waves were filtered. Second, once a household was successfully reached, an eligible family member with the soonest next birthday was invited to the survey. All telephone interviews were conducted by trained interviewers from the Public Opinion Program at the University of Hong Kong, a reputable local survey agency. Among 5,773 invited respondents, 4,054 were successfully interviewed (response rate = 70.2%). A randomly selected subset of 687 (17.0%) completed the Chinese version of FCS [61.1% female; mean age [standard deviation (SD)] 56.6 (19.1) years; 42.8% had secondary educational attainment]. We evaluated the factor structure by exploratory factor analysis (EFA), internal consistency reliability, and construct validity of the Chinese version of FCS. We also examined associations of sociodemographic characteristics and family communication methods with positive family communication.

#### Study 2: Happy Family Kitchen Movement Project

A total of 54 social service units and schools collaborated with the research team to design and implement the trial in 1,983 participants (76.7% female; 57.8% aged 20–59 years; 52.3% had secondary educational attainment) from 1,467 families in all 18 districts in Hong Kong. The social service units and schools were randomly allocated as clusters with the participants they recruited into three groups. Positive Physical Activity group (PPA; intervention arm 1) and Positive Healthy Diet group (PHD; intervention arm 2) received a core session of about 2 h, followed by a booster session of about 1 h a month later. The control group (the waitlist control arm) received a tea gathering session at the beginning and a month later. The core session in the PPA group included group activities and homework assignments focusing on positive psychology and physical activity. The core session in the PHD group focused on positive psychology and healthy diet. The booster session in the PPA and PHD groups focused on consolidating knowledge, skills, and experience gained from the core sessions. The tea gathering sessions in the control group included activities unrelated to PPA/PHD. The participants completed assessment questionnaires at four time points: baseline (T1), immediately post-intervention (T2), 1-month (T3) follow-up, and 1-month follow-up (T4). We conducted a confirmatory factor analysis (CFA) to determine the replicability of the EFA results in Study 1. We also evaluated the 1-month test–retest reliability and construct validity of the Chinese version of FCS.

### Measurements

The translation process of FCS followed the guidelines provided by the author of the original FCS (Olson and Barnes, 2004). A translation team was created and comprised of professional translators who are bilingual in English and Chinese. The FCS was first translated into traditional Chinese and then back-translated into English until a consensus was achieved. Examples of FCS items are “Family members are satisfied with how they communicate with each other,” “Family members are very good listeners,” and “Family members express affection to each other” (Olson and Barnes, 2004). Each item scores on a five-point Likert scale ranging from 1 = strongly disagree to 5 = strongly agree. A higher total score (range 10–50) indicates a greater level of positive family communication.

We examined the construct validity of FCS using the following measurements. In Study 1 and Study 2, Subjective Happiness Scale (SHS; range 1–7; Cronbach’s alpha 0.75 in Study 1 and ranged 0.72–0.75 in Study 2) has four items to measure individual happiness (Lyubomirsky and Lepper, 1999; Nan et al., 2014). Family Well-being Scale has three single items (each range 0–10) on family health, harmony, and happiness that were developed specifically in Chinese culture (Chan et al., 2011; Lam et al., 2012). The single item of family happiness has been validated in Hong Kong (Shen et al., 2019). Different measurements between Study 1 and Study 2 can broaden the scope of examination and reduce questionnaire length and response burden. We used measurements that were only included in Study 1 for additionally examining the convergent validity of FCS: Family APGAR Scale (range 0–10; Cronbach’s alpha 0.86) has five items to measure the family functioning (Smilkstein, 1978; Chan et al., 1988). Respondents were asked whether they had sufficient communication with family members on a five-point scale ranging from 1 = very insufficient, 2 = insufficient, 3 = fair, 4 = sufficient, to 5 = very sufficient. Perceived family communication quality was rated on an 11-point scale, where 0 = very poor, 5 = half-half, and 10 = very good. Short Form-12 Health Survey Version 2 (SF-12) only included in Study 2 was used for examining discriminative validity. SF-12 has 12 items to measure physical and mental health-related quality of life (HRQoL; Cronbach’s alpha ranged 0.79–0.81 for PCS and 0.75–0.76 for MCS) (Ware et al., 1996; Lam et al., 2005). The raw scores are transformed into the Physical Component Subscale (PCS; range 0–100) and Mental Component Subscale (MCS; range 0–100).

Respondents in Study 1 were asked as to how often they used the following methods to communicate/chat with family members, including face to face, phone calls, IM (e.g., WhatsApp), SNS (e.g., Facebook), video calls (e.g., Skype, FaceTime, WeChat video call), and email. Responses included often, sometimes, seldom, and never. We dichotomized the frequency as never/seldom (reference) vs. sometimes/often due to the skew distribution of the continuous variable.

Sociodemographic characteristics included gender, age, marital status, employment status, educational attainment, and monthly household income.

### Statistical Analysis

#### Study 1: The Hong Kong Family and Health Information Trends Survey

All data were weighted by gender, age, and educational attainment distribution of the Hong Kong general population using the random iterative method (Izrael et al., 2004). Missing data were handled by the available case analyses as there were minimal missing values for all variables (<2.5%). Preliminary analyses were conducted to ensure the appropriateness for an EFA: (1) the normality of FCS score was confirmed with a skewness value of −0.65 (≤ | 2.0|) and a kurtosis value of 4.19 (≤ | 7.0|), given the large sample size (Kim, 2013); (2) flooring and ceiling effects were not present, with 0.15 and 4.62% (both ≤ 15%) respondents had the lowest or highest possible FCS score (Terwee et al., 2007); (3) the Kaiser–Meyer–Olkin measure of sampling adequacy was of 0.935 and the Bartlett test of sphericity reached a significant level (χ^2^ = 3272.453, df = 45, *P* < 0.001), indicating the strong correlations among FCS items for an EFA.

The EFA extracted factors from the 10 items of FCS using the principal factor method with promax rotation (i.e., an oblique rotation that allows for correlations between factors). The factor structure was determined by multiple approaches: Kaiser’s criterion (eigenvalues >1), scree plot, parallel analysis with principal components and 10,000 random datasets (the larger the number, the more accurate the estimate; Dinno, 2009); and the minimum average partial test (Courtney and Gordon, 2013). Parallel analysis and the minimum average partial test have been suggested to be the most accurate of all the approaches (Velicer et al., 2000), and consistent results would increase the confidence of the factor structure of FCS. Factors were retained with the adjusting eigenvalues (accounting for sampling bias) greater than that could be generated from random data in parallel analysis and with the minimum average squared partial correlations. Factor loadings were evaluated by the following criteria: ≥ 0.71 excellent, 0.63–0.70 very good, 0.55–0.62 good, 0.45–0.54 fair, 0.32–0.44 poor, and < 0.32 unacceptable (Comrey and Lee, 2013).

Internal consistency reliability was determined by Cronbach’s alpha and McDonald’s Omega coefficient, requiring value of ≥ 0.70 acceptable, ≥ 0.80 good (Terwee et al., 2007; Hayes and Coutts, 2020). The convergent validity was determined by the correlations of FCS score with scores of SHS, family health, family harmony, family happiness, family APGAR, and perceived sufficiency, and quality of family communication. Pearson’s correlation coefficients (*r*) were calculated, and values of | *r*| were evaluated by the following criteria: 0.68–1 strong, 0.36–0.67 moderate, and 0–0.35 weak (Taylor, 1990). Differences in *r* were assessed using Fisher z-transformation test. Known-group validity was evaluated by comparing the mean FCS scores by sociodemographic characteristics and communication methods using linear regression analyses. Multivariable analyses were used to test whether the differences can still present after mutual adjustments. We hypothesized higher FCS scores observed in people with higher household income based on similar associations reported in our qualitative interviews (Chan et al., 2011; Lam et al., 2012). Frequent face to face and ICTs use for family communication have been associated with improved family well-being, which may increase FCS scores (Wang et al., 2015; Shen et al., 2017b). We accordingly hypothesized higher FCS scores observed in people having frequent family communication through face to face and ICTs. Analyses were conducted on Stata 15.0. A *P*-value of < 0.05 was considered statistically significant.

#### Study 2: Happy Family Kitchen Movement Project

The principle of intention-to-treat analysis was adopted. CFA with diagonally weighted least squares estimation for ordinal data was performed to examine the factor structure identified by EFA in Study 1 (Li, 2016). The adequacy of model fit was determined by a combination of the following indices: relative/normed Chi-square statistic (χ^2^/df, < 3), goodness-of-fit index (GFI; ≥ 0.95), comparative fit index (CFI; ≥ 0.90), root mean square error of approximation (RMSEA; < 0.06), root mean square residual (RMR; < 0.08), and standardized RMR (SRMR; < 0.08) (Hooper et al., 2008; Kline, 2015). We reported results of Chi-square test for descriptive purpose but not for evaluating the model fit (cutoff for good fit: *P* > 0.05), because the result is always statistically significant in large samples (Hooper et al., 2008).

One-month test–retest reliability was determined by intraclass correlation coefficients in the control group, calculated based on a consistency two-way mixed-effects model, by the following criteria: 0.90–1 excellent, 0.75–0.89 good, 0.50–0.74 moderate, and 0–0.49 poor (Koo and Li, 2016). Convergent validity was determined by correlations of FCS score with SF-12 PCS and MCS, SHS, family health, family harmony, and family happiness. Discriminant validity was determined by differences between the correlation of FCS score with SF-12 PCS and that with MCS. Pearson’s correlation coefficients (*r*) were calculated, and the values of | *r*| were evaluated by the following criteria: 0.68–1 strong, 0.36–0.67 moderate, and 0–0.35 weak (Taylor, 1990). Partial correlation analysis was used to account for the intervention effect at T3 and T4. Differences in *r* were assessed using Fisher z-transformation test. Analyses were conducted on SPSS 25.0 except for CFA on LISREL 11. A *P*-value of < 0.05 was considered statistically significant.

## Results

### Factor Structure and Psychometric Properties of Chinese Version of Family Communication Scale

**FIGURE 1:**
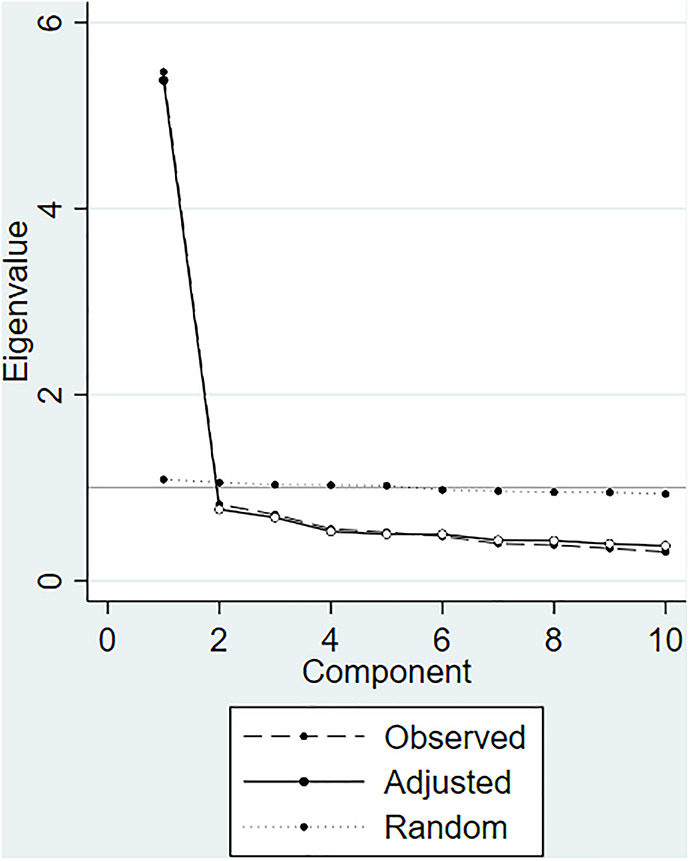
Parallel analysis for determining the number of factors to retain for the exploratory factor analysis in the population-based sample (*N* = 687).

In the population-based sample of Study 1, EFA showed that a one-factor structure should be retained: (1) Only the first factor (eigenvalue = 4.97) met the Kaiser’s criterion and explained 86.72% of the variance; (2) visual examination of screen plot indicated that the first factor accounted for most of the variance (the dashed line in Figure 1); (3) parallel analysis indicated that the adjusted eigenvalue (the solid line in Figure 1) of the first factor was greater than that could be obtained in random data (the dotted line in Figure 1); (4) the minimum average partial test indicated the minimum average squared partial correlations of the first factor (0.021 in Table 1). All 10 items had good-to-excellent factor loadings (range 0.55–0.79) (Table 2). The internal consistency was good (Cronbach’s alpha = 0.91; McDonald’s Omega = 0.91). Removal of any item yielded a Cronbach’s alpha ranging 0.89–0.91.

**TABLE 1:** The minimum average partial test for determining the number of factors to retain for the exploratory factor analysis in the population-based sample (*N* = 687).

**Number of factors**	**Average squared partial correlations**
0	0.25
1	0.021
2	0.039
3	0.057
4	0.093
5	0.12
6	0.19
7	0.29
8	0.46
9	1

**TABLE 2:** Descriptive statistics, factor loadings, and internal consistency of the Chinese version of the Family Communication Scale in the population-based sample (*N* = 687).

**FCS item^a^**	**Mean score (SD)^b^**	**Factor loading^c^**	**Corrected item-total correlation**	**Cronbach’s alpha if item deleted^d^**
1	3.77 (0.89)	0.70	0.67	0.89
2	3.68 (0.96)	0.70	0.66	0.90
3	3.93 (0.85)	0.75	0.72	0.89
4	3.85 (0.90)	0.70	0.66	0.90
5	3.76 (0.92)	0.74	0.70	0.89
6	3.82 (0.85)	0.75	0.72	0.89
7	3.90 (0.79)	0.68	0.64	0.90
8	3.79 (0.84)	0.79	0.75	0.89
9	3.41 (1.03)	0.55	0.53	0.91
10	3.84 (0.86)	0.64	0.60	0.90

*^a^Each item scores from 1 = “strongly disagree” to 5 = “strongly agree.”*

*^b^Weighted by age, gender, and educational attainment distribution of the Hong Kong general population.*

*^c^Proportion of total variance = 86.72%.*

*^d^Overall Cronbach’s alpha = 0.91; McDonald’s Omega = 0.91.*

**FIGURE 2:**
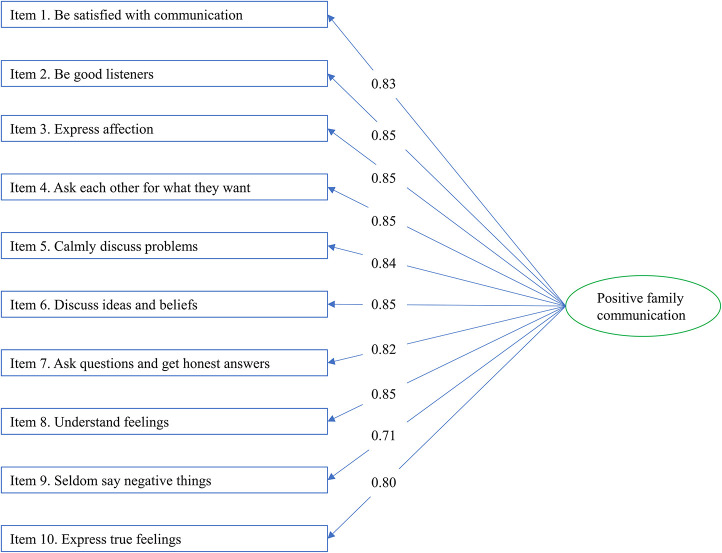
The final one-factor model of the Family Communication Scale indicated by the confirmatory factor analysis with standardized path coefficients in the community-based sample (*N* = 1983).

CFA was performed on the one-factor structure in the community-based sample of Study 2. All model fit indices were within the prespecified cut-off values (GFI = 0.998 > 0.95, CFI = 0.994 > 0.90, RMSEA = 0.044 < 0.06, RMR = 0.016 < 0.08, SRMR = 0.028 < 0.08), except for χ^2^/df = 4.61 > 3 (Table 3). No further modifications were made and the final model is presented in Figure 2.

**TABLE 3:** Fit statistics for the one-factor model of the Chinese version of the Family Communication Scale in the community-based sample (*N* = 1,983).

**Model fit indices with cutoff [36,37]**	**χ^2^ (*P* > 0.05)**	**Df**	**χ^2^/df**	**GFI ≥ 0.95**	**CFI ≥ 0.90**	**RMSEA < 0.06**	**RMR < 0.08**	**SRMR < 0.08**
With one factor and 10 test items ^a^	161.22 (<0.001)	35	4.61	0.998	0.994	0.044 (0.048, 0.090)	0.016	0.028

*χ^2^, Chi-square; df, degrees of freedom; CFI, comparative fit index; RMSEA, root mean square standard error of approximation; RMR, root mean square residual; SRMR, standardized root mean square residual.*

*^a^The one-factor structure was based on the result of exploratory factor analysis.*

The intraclass correlation coefficient for test–retest reliability over 1 month was 0.69 (*P* < 0.001) in the community-based sample of Study 2. The FCS score was positively and moderately correlated with the scores of SHS, family health, family harmony, family happiness, family APGAR, and perceived family communication sufficiency and quality (*r* range 0.40–0.60; all *P* < 0.001) in Study 1 (Table 4). The correlation of the FCS score with perceived family communication quality (*r* = 0.60) was significantly stronger than that with perceived family communication sufficiency (*r* = 0.40) (*P* < 0.001). Family communication was also positively and moderately correlated with scores of SHS, family health, family harmony, and family happiness at baseline (T1), 1-month follow-up (T3), and 3-month follow-up (T4) (all *P* < 0.001), regardless of the intervention effects in Study 2. The correlations of the FCS score with SF-12 MCS (*r* range 0.31–0.34) were significantly stronger than those with PCS (*r* range 0.13–0.19) at all three time points (*P* < 0.001), regardless of the intervention effects.

**TABLE 4:** Correlations of the Family Communication Scale score with scores of 12-item Short Form Health Survey Version 2, Subjective Happiness Scale, family health, family harmony, family happiness, family APGAR scale, and perceived family communication quality and sufficiency in the population-based sample and community-based sample.

**Correlation with the Family Communication Scale score (FCS; range 10–50)^a^**	**Population-based sample (*N* = 687)**	**Community-based sample (*N* = 1983)**		
		**Baseline (T1)**	**1-Month follow-up (T3)^b^**	**3-Month follow-up (T4)^b^**
SF-12 Physical Component Subscale (PCS; range 0–100)	−	0.13	0.19	0.17
SF-12 Mental Component Subscale (MCS; range 0–100)	−	0.31	0.34	0.34
Subjective happiness scale (SHS; range 1–7)	0.43	0.47	0.48	0.46
Family health (range 0–10)	0.47	0.51	0.50	0.48
Family harmony (range 0–10)	0.56	0.61	0.59	0.58
Family happiness (range 0–10)	0.57	0.58	0.58	0.56
Family APGAR Scale (range 0–10)	0.49	−	−	−
Perceived family sufficiency (range 1–5)	0.40	−	−	−
Perceived family quality (range 0–10)	0.60	−	−	−

*SF-12, 12-item Short Form Health Survey Version 2; APGAR, Adaption, Partnership, Growth, Affection, Resolve.*

*^a^All P-values for Pearson correlation coefficients < 0.001.*

*^b^Partial correlation was used to account for the intervention effect when assessing the correlations.*

### Associations of Sociodemographic Characteristics With Positive Family Communication

The mean FCS score (SD) was 37.8 (6.2) in the population-based sample (Table 4). Multivariable linear regression analyses showed that housekeepers had higher FCS scores (adjusted β = 3.57, 95% CI 0.23, 6.92), adjusting for other sociodemographic characteristics. Monthly household income was positively associated with FCS scores (*P* for trend = 0.045) (Table 5).

**TABLE 5:** Associations of sociodemographic characteristics with Family Communication Scale score in the population-based sample (*N* = 687).

	***n* (%)^a^ (*N* = 693)**	**Mean FCS score (SD)^a^,^b^**	**Crude β (95% CI)**	**Adjusted^c^ β (95% CI)**
**Gender**				
Male	309 (44.5)	37.7 (5.5)	0	0
Female	385 (55.5)	37.9 (6.8)	0.26(−0.99,1.50)	−0.24(−1.58,1.10)
**Age, years**				
18–24	66 (9.5)	37.3 (7.1)	0	0
25–44	233 (33.6)	36.7 (3.5)	−0.59(−2.77,1.59)	−1.80(−4.51,0.91)
45–64	270 (38.9)	38.4 (6.2)	1.13(−0.91,3.17)	−0.19(−3.02,2.64)
≥65	125 (18.0)	38.8 (9.3)	1.48(−0.52,3.47)	0.29(−2.80,3.39)
*P* for trend			0.007	0.096
**Marital status**				
Unmarried	172 (24.8)	36.8 (6.0)	0	0
Cohabitated/married	460 (66.4)	38.2 (5.9)	1.35(−0.22,2.92)	0.25(−1.96,2.45)
Divorced/separated/widowed	61 (8.8)	37.7 (7.7)	0.85(−1.22,2.92)	−0.16(−2.94,2.62)
**Employment status**				
Unemployment	39 (5.6)	34.4 (3.5)	0	0
In-paid employment	328 (47.3)	37.6 (5.0)	3.26(0.43,6.08)	2.55(−0.40,5.50)
Retired	152 (22.0)	38.5 (8.2)	4.15(1.32,6.97)**	2.67(−0.66,6.00)
Housekeeper	134 (19.3)	38.8 (6.1)	4.43(1.45,7.41)**	3.57(0.23,6.92)*
Full-time student	41 (5.9)	36.8 (8.2)	2.47(−1.27,6.21)	0.76(−3.37,4.90)
**Educational attainment**				
Primary or below	171 (24.6)	38.6 (6.5)	0	0
Secondary	345 (49.7)	37.2 (5.3)	−1.38(−2.94,0.17)	−0.51(−2.16,1.14)
Tertiary	178 (25.6)	38.3 (7.4)	−0.32(−2.01,1.36)	0.36(−1.71,2.42)
*P* for trend			0.748	0.863
**Monthly household income (HK $)^d^**				
≤19,999	237 (34.2)	37.0 (6.2)	0	0
20,000–29,999	131 (18.8)	37.5 (6.3)	0.42(−1.65,2.48)	0.42(−1.57,2.41)
30,000–39,999	92 (13.3)	37.9 (6.1)	0.90(−1.08,2.89)	1.37(−0.55,3.29)
≥40,000	176 (25.4)	38.4 (5.3)	1.38(−0.03,2.79)	1.42(−0.21,3.05)
*P* for trend			0.052	0.045
Unstable/refused to answer	58 (8.3)	39.5 (7.7)	2.41(0.54,4.27)*	1.86(−0.14,3.85)

*FCS, Family Communication Scale, range 10–50.*

**P < 0.01; *P < 0.05.

*^a^Weighted by age, gender, and educational attainment distribution of the Hong Kong general population.*

*^b^Mean FCS score (SD) = 37.8 (6.2).*

*^c^Mutually adjusted for other variables in the table.*

*^d^US$ 1 = HK$ 7.8.*

### Associations of Communication Method With Positive Family Communication

The most frequent method of family communication was face to face (92.0%), followed by phone calls (66.7%) and IM (59.4%) in the population-based sample (Table 6). Frequent face-to-face communication was strongly associated with higher FCS scores (adjusted β = 6.33, 95% CI 3.71, 8.96). Frequent use of ICTs including phone calls (adjusted β = 2.74, 95% CI 1.50, 3.97), SNS (adjusted β = 2.26, 95% CI 0.55, 3.96), and IM (adjusted β = 1.91, 95% CI 0.53, 3.29) were associated with higher FCS scores. Among the respondents who frequently conducted face-to-face family communication, higher FCS scores were observed for frequent use of phone calls (adjusted β = 1.99, 95% CI 0.82, 3.15) and SNS (adjusted β = 1.84, 95% CI 0.13, 3.57).

**TABLE 6:** Associations of communication method with Family Communication Scale score in the population-based sample (*N* = 687).

	*n* (%)	Mean FCS score (SD)^a^,^b^	Crude β (95% CI)	Adjusted^c^ β (95% CI)	Adjusted^c^ β (95% CI) (*n* = 628)^d^
**Face to face**					
Never/seldom	55 (8.0)	32.1 (8.4)	0	0	−
Sometimes/often	628 (92.0)	38.3 (5.7)	6.23 (3.57, 8.89)***	6.33 (3.71, 8.96)***	−
**Phone call**					
Never/seldom	227 (33.3)	36.0 (7.0)	0	0	0
Sometimes/often	455 (66.7)	38.7 (5.6)	2.65 (1.34, 3.96)***	2.74 (1.50, 3.97)***	1.99 (0.82, 3.15)***
**Instant messaging (e.g., WhatsApp)**					
Never/seldom	277 (40.6)	37.0 (7.0)	0	0	0
Sometimes/often	405 (59.4)	38.4 (5.5)	1.38 (0.09, 2.66)*	1.91 (0.53, 3.29)**	1.18 (-0.25, 2.61)
**Video call (e.g., Skype, FaceTime, WeChat video call)**					
Never/seldom	555 (81.4)	37.6 (6.5)	0	0	0
Sometimes/often	127 (18.6)	38.6 (4.6)	0.92 (−0.49, 2.34)	1.10 (−0.26, 2.45)	0.49 (−0.87, 1.86)
**Social networking sites (e.g., Facebook)**					
Never/seldom	595 (87.2)	37.5 (6.3)	0	0	0
Sometimes/often	87 (12.8)	39.5 (5.3)	1.95 (0.34, 3.57)*	2.26 (0.55, 3.96)**	1.84 (0.13, 3.57)*
**Email**					
Never/seldom	626 (91.8)	37.7 (6.2)	0	0	0
Sometimes/often	56 (8.2)	39.1 (5.5)	1.40 (−0.19, 2.99)	1.10 (−0.51, 2.72)	0.63 (−1.00, 2.26)

*FCS, Family Communication Scale, range 10–50.*

***P < 0.001; **P < 0.01; *P < 0.05.

*^a^Weighted by age, gender, and educational attainment distribution of the Hong Kong general population. ^b^Mean FCS score (SD) = 37.8 (6.2).*

*^c^Adjusted for gender, age, marital status, employment status, educational attainment, and monthly household income.*

*^d^Among respondents who frequently conducted face-to-face family communication.*

## Discussion

The EFA showed a one-factor model of the Chinese version of FCS that comprises all 10 items in the population-based sample. The model was replicated by CFA in the community-based sample. Results of relative/normed Chi-square statistic (χ^2^/df = 4.61) was higher than the cutoff of 3 (Kline, 2015). However, various cutoffs of χ^2^/df ranging 2–5 have been used in the literature, and no consensus was found (Tabachnick et al., 2007). Other model fit indices including GFI, CFI, RMSEA, RMR, and SRMR were within the prespecified cutoff values. Taken together, the one-factor model suggested was acceptable. The one-factor structure was also identified in the original scale (Olson and Barnes, 2004) and the Portuguese validation (Gomes et al., 2017). In contrast, the Spanish validation in the Chilean population showed a two-factor structure, suggesting the independence of emotional/affective dimension of family communication and the other dimension related to more general communication skills, such as problem-solving skills and listening skills (Rivadeneira and López, 2017). The Turkish validation showed a one-factor structure but discarded items on self-disclosure and affective communication because of the low factor loadings and the tendency to be under another dimension (Türkdoğan et al., 2018). This can be a reflection of listening centeredness and implicit communication style valued in the collectivist Asian cultures (Bond, 2010). Our findings of retaining all 10 items contrasted with this notion, suggesting a more direct exchange of information both factual and emotional within the Hong Kong Chinese population. Similar preference of explicit communication style was reported in our previous qualitative study (Lam et al., 2012). One possible explanation is that the Western media influences and the busy urban life have encouraged more direct and explicit communication in Hong Kong (Lam et al., 2012), the most westernized and modernized city in China.

Our results supported the FCS as a reliable and valid measurement of positive family communication in the Chinese population. The internal consistency reliability (Cronbach’s alpha = 0.91; McDonald’s Omega = 0.91) was good and comparable to those obtained in the original scale and other validations (Cronbach’s alpha 0.88–0.92) (Olson and Barnes, 2004; Smith et al., 2009; Martínez-Pampliega et al., 2017; Rivadeneira and López, 2017). The 1-month test–retest reliability (intraclass correlation coefficient = 0.69) was moderate, despite the potential effects of tea gathering sessions on family communication in the control group. The correlation of positive family communication with mental HRQoL was significantly stronger than that with physical HRQoL. Although not directly measuring family communication, our previous study showed a stronger correlation of family happiness with mental HRQoL than physical HRQoL (Shen et al., 2019). Positive family communication was found to be positively and moderately correlated with individual happiness measured by SHS (Lyubomirsky and Lepper, 1999), family functioning measured by family APGAR (Smilkstein, 1978), and family well-being including health, harmony, and happiness developed specifically in the Chinese culture (Chan et al., 2011; Lam et al., 2012). The correlation of positive family communication with perceived communication quality was statistically stronger than that with perceived sufficiency. The difference implied that quality enhancement might be more important than the increase in family time to develop positive communication skills, particularly in Hong Kong where long work hours challenge shared family time (Wharton and Blair-Loy, 2006; Ho et al., 2018). A study among romantic partners also supported communication quality indicators (e.g., depth, smooth, social) but not quantity on predicting intimacy and relational satisfaction (Emmers-Sommer, 2004).

A higher household income was associated with greater positive family communication. High-income families tend to experience fewer financial problems particularly monetary difficulties that could induce stress and family conflicts (Orthner et al., 2004). Alternatively, people with high income were more likely to seek or share knowledge and skills to enhance family communication because of more social support, cognitive skills, and information literacy, which are documented barriers for people with low socioeconomic status (Wang et al., 2014; Shen et al., 2017b).

Frequent use of ICTs including phone calls, SNS, and IM with family members was associated with greater positive family communication. The perpetual connectivity pattern represented by ICTs can facilitate family communication in real time and/or be conducted asynchronously. For example, family members could take advantage of the mobility and immediacy to coordinate family activities through mobile devices during time on waiting or on the move (Lanigan, 2009). The pattern of media multiplexity by ICTs allows both factual and emotional information to be exchanged in a diversity of media such as texts, pictures, audio clips, and videos (Carvalho et al., 2015). Communication through phone calls could further provide instant feedback and multiple cues such as tones and inflection. Studies also suggested the potential adverse effects of ICTs on family communication, as ICTs might reduce communication content and context compared with the traditional face-to-face method that conveys verbal, non-verbal, and tacit knowledge simultaneously (Carvalho et al., 2015). We found that frequent face-to-face communication was strongly associated with positive family communication, which was consistent with our previous findings of the central role of face-to-face communication in improving family well-being (Wang et al., 2015; Shen et al., 2017b). Among the respondents who frequently conducted face-to-face family communication, frequent use of phone calls, and SNS were associated with even greater levels of positive family communication. Such findings supported that ICTs could be utilized as a supplement and extension to the traditional face-to-face communication method.

One of the limitations of Study 1 was the cross-sectional study design, which was subjected to residual confounding and restricted the inference of temporal sequence of the observed associations. We used the landline telephone sampling method, which excluded the increasing mobile phone-only households. The effects of non-response bias and coverage bias on the observed associations are uncertain. However, data were weighted according to gender, age, and educational attainment distribution of Hong Kong general population to increase the representativeness. We did not assess the geographical distance between family members, which could influence the selection of the communication method and frequency of use (Carvalho et al., 2015). All scales used in Study 1 and Study 2 were self-reported, which could be subjected to bias. Samples were from the Hong Kong Chinese population who have been exposed to social modernization, urban living, and Western cultural influences. Generalizability to rural settings and Chinese communities outside Hong Kong needs to be further studied. For example, measurement invariance tests of FCS can be used to assess the differences between urban and rural settings. Content validity and responsiveness of FCS were not evaluated. However, FCS scores increased with sustainable small effects up to 12 weeks in our previous interventions for enhancing family communication and well-being (Ho et al., 2016a,b).

Our study suggests several avenues for future research. The stronger correlation of positive family communication with perceived family communication quality than sufficiency warranted qualitative research on content and context of communication among family members to provide a deeper understanding of the important role of communication quality. Longitudinal studies are needed to distill the causal relations between communication quality and positive family communication. Income inequalities in positive family communication warranted intervention studies for enhancing family communication specifically in low-income families. Our study is the first to show the ability of FCS to distinguish people having frequent family communication using ICTs, and ICTs could enhance family communication as supplements and extension of the traditional face-to-face method. The findings inform future digital health research in the family context that FCS can be an appropriate outcome measure.

## Conclusion

We identified the one-factor structure of the Chinese version of FCS, which can serve as a valid and reliable measurement of positive family communication in the Chinese population. A higher monthly household income and frequent use of face-to-face communication and ICTs including phone calls, IM, and SNS were associated with greater positive family communication. Frequent use of phone calls and SNS can improve positive family communication among people who frequently conducted face-to-face family communication. The findings indicated that ICTs could be utilized as a supplement for traditional face to face to enhance family communication in the Chinese population.

## Data Availability Statement

The datasets presented in this article are not readily available because the data that support the findings of this study are available from the FAMILY project but restrictions apply to the availability of these data, which were used under license for the current study, and so are not publicly available. Data are however available from the authors upon reasonable request and with permission of the Hong Kong Jockey Club Charities Trust. Requests to access the datasets should be directed to jcfamily@hku.hk.

## Ethics Statement

Ethical approvals of Study 1 and Study 2 were granted by the Institutional Review Board of the University of Hong Kong/Hospital Authority Hong Kong West Cluster. Verbal informed consent was obtained from the respondents in Study 1. Written consent was obtained from the adult participants in Study 2. For participants younger than 18 years in Study 2, written consent was obtained from the next of kin, caretakers, or guardians on their behalf.

## Author Contributions

MW, KV, SC, and THL conceived the study. NG analyzed the data from Study 1 and wrote the first draft of the manuscript. HH analyzed the data from Study 2. NG, HH, MW, and TTL interpreted the data. All authors critically revised and approved the final version of the manuscript.

## Conflict of Interest

The authors declare that the research was conducted in the absence of any commercial or financial relationships that could be construed as a potential conflict of interest.

## Publisher’s Note

All claims expressed in this article are solely those of the authors and do not necessarily represent those of their affiliated organizations, or those of the publisher, the editors and the reviewers. Any product that may be evaluated in this article, or claim that may be made by its manufacturer, is not guaranteed or endorsed by the publisher.
